# Cross-Sectional Analysis of IL-6, TNF-α, Adiponectin, Leptin, and Klotho Serum Levels in Relation to BMI Among Overweight and Obese Children Aged 10–14 in La Rioja, Spain

**DOI:** 10.3390/children12010089

**Published:** 2025-01-14

**Authors:** Beatriz Fernández-Vallejo, Francisco Jiménez Monteagudo, Lourdes Romero, Maria Isabel López Aznárez, María del Carmen Romero Cobas, Laura Pérez-Martínez

**Affiliations:** 1 Servicio de Pediatría, Hospital Universitario San Pedro, 26006 Logroño, Spain; 2 Pediatría de Atención Primaria, Centro de Salud Espartero, 26006 Logroño, Spain; fjimenezm@riojasalud.es; 3 Centro de Investigación Biomédica de La Rioja (CIBIR), 26006 Logroño, Spain; lrbarruete@riojasalud.es (L.R.); lperez@riojasdalud.es (L.P.-M.); 4 Pediatría de Atención Primaria, Centro de Salud Guindalera, 26006 Logroño, Spain; ilaznares@riojasalud.es; 5 Medicina Familiar y Comunitaria, Centro de Salud Gonzalo de Berceo, 26006 Logroño, Spain; mcromero@riojasalud.es

**Keywords:** adipocytokines, children, leptin, leptin/adiponectin ratio, obesity, overweight, sex

## Abstract

Background: Childhood obesity is a major public health concern, being linked to an increased risk of metabolic disorders and cardiovascular disease. Even in childhood, obesity is associated with systemic low-grade inflammation, which is a critical factor in the development of atherosclerosis and a predictor of cardiovascular morbidity and mortality. Objectives: To describe the prevalence of obesity and examine the relationship between IL-6, TNF-α, adiponectin, leptin, the leptin/adiponectin (L/A) ratio, and Klotho levels with BMI in children. Methods: This cross-sectional study included children aged 10–14 years from La Rioja, Spain. Participants were selected based on BMI criteria for overweight (85th–95th percentiles) and obesity (>95th percentile). Socio-demographic and anthropometric data and blood samples were collected and analyzed for IL-6, TNF-α, adiponectin, leptin, and Klotho. Results: A total of 340 participants were included, with 276 (81.2%) classified as normal weight and 64 (18.8%) as overweight or obese. Mean age was similar between groups (*p* = 0.40). Obesity was more prevalent in males (59.4%, *p* = 0.048). Obese participants had higher mean birth weight (*p* = 0.003), current height (*p* = 0.04), BMI (*p* < 0.0001), and abdominal circumference (*p* < 0.0001). BMI correlated positively with leptin (r = 0.54, *p* = 0.0008) and the L/A ratio (r = 0.40, *p* = 0.025), showing sex-specific differences. Conclusions: This study underscores leptin and the L/A ratio as potential biomarkers of metabolic dysregulation in childhood obesity, particularly in females. Longitudinal studies are needed to confirm these findings and assess the clinical utility of these biomarkers in pediatric obesity management.

## 1. Introduction

Overweight and obesity in children and adolescents are global public health concerns [1]. In 2015, the Global Burden of Disease Obesity Collaborators reported a 5.0% global prevalence of childhood obesity in 2015, affecting 107.7 million children [2]. The World Obesity Federation estimates this trend will continues to rise, with 158 million children aged 5–19 years affected in 2020, 206 million by 2025, and 254 million by 2030 [3]. A recent systematic review and meta-analysis (2000–2023) reported prevalences of 8.5% for obesity, 14.8% for overweight, and 22.2% for excess weight, with notable regional variations, highlighting the need for context-specific interventions [4]. In many regions, childhood obesity now surpasses malnutrition as a more pressing health issue [5], and its strong link to adult obesity underscores the urgency of prevention [6,7]. Children with obesity face a significantly higher risk of developing metabolic disorders, such as type 2 diabetes mellitus, atherosclerosis, stroke, sleep apnea, respiratory dysfunctions, and certain cancers [6,7]. This growing epidemic poses not only significant health risks but also increasing economic burdens for individuals and society [8].

Obesity is associated with the presence of prothrombotic factors (e.g., fibrinogen, homocysteine), inflammatory markers (e.g., interleukin-6 (IL-6), tumor necrosis factor-alpha (TNF-α), C-reactive protein (CRP)), and adipocytokines (e.g., leptin, adiponectin)) [9]. These biomarkers are regarded as indicators of cardiovascular and metabolic disease risk [10]. Even during childhood, obesity has been associated with the presence of a systemic low-grade inflammatory state [11,12]. This chronic low-grade inflammation plays a pivotal role in the development of atherosclerosis [13], and serves as a predictor of cardiovascular morbidity and mortality [14,15].

Adipose tissue inflammation is a significant contributor to systemic inflammation, which is closely associated with insulin resistance. This mechanism increases the risk of type 2 diabetes mellitus, metabolic syndrome, and nonalcoholic fatty liver disease, all of which contribute to future cardiovascular risk [16]. Among the adipocytokines, leptin and adiponectin are particularly relevant to metabolic syndrome [10]. Leptin, one of the most abundant adipokines secreted by adipocytes, regulates energy balance, the hypothalamic–pituitary–adrenal axis, and immune function [17,18]. Elevated leptin levels, proportional to body fat stores, are commonly observed in individuals with obesity [19,20]. Leptin also promotes vascular dysfunction by contributing to hypertension, angiogenesis, and atherosclerosis [10]. In contrast, adiponectin plays an anti-inflammatory, anti-atherogenic, and insulin-sensitizing effects, providing protection against type 2 diabetes and cardiovascular disease [10,21]. The leptin-to-adiponectin (L/A) ratio has emerged as a sensitive biomarker for early metabolic dysregulation and may offer superior diagnostic utility compared to leptin or adiponectin alone [22].

Klotho, a complex hormonal regulator secreted primarily by the kidneys [23], has also gained attention for its role in whole-body metabolism. It enhances insulin sensitivity [24], insulin secretion [25], and stimulates lipid oxidation [26]. Reduced circulating levels of Klotho have been reported in individuals with diabetes [27], highlighting its potential as a marker of metabolic heath. To date, the studies evaluating this marker in adolescents are limited [28,29].

This study analyzed the prevalence of overweight and obesity in a pediatric population. Recognizing the increasing importance of low-grade inflammation in overweight and obese children, we also investigated the relationship between IL-6, TNF-α, adiponectin, leptin, the leptin/adiponectin ratio, and Klotho levels with body mass index (BMI). Understanding these associations is crucial for uncovering the mechanisms underlying obesity-related metabolic dysregulation in children and adolescents.

## 2. Material and Methods

### 2.1. Ethical Committee

This study was evaluated and approved by the Ethical and Clinical Research Committee of the Comunidad Autonoma de La Rioja (CEImLAR), who acted as a safeguard of the rights and well-beings of the participants in the clinical research, ensuring that these projects are carried out in an ethical, responsible manner and with the maximum scientific rigor.

### 2.2. Study Design

This cross-sectional study focused on children aged 10 to 14 years, using data from La Rioja Health Card system, which identified 11,473 individuals in this range in 2018. The required sample size for a population of 11,473 individuals, with an estimated prevalence range of 32.3% to 35.3% (mean 33.8%) [30], considering a 95% confidence level and a 5% margin of error, is approximately 334 individuals.

### 2.3. Patient Selection Criteria

Inclusion criteria included children aged 10 to 14 years of both sexes attending either scheduled periodic check-ups as part of the “Healthy Child Program” or unscheduled consultations for other reasons. Signed informed consent from parents and/or legal guardians was required. Children with severe cognitive impairment (e.g., cerebral palsy, autism spectrum disorders, organic neurological conditions), neuromuscular disorders, oncological conditions, psychiatric illness, chronic inflammatory or endocrine disorders, acute illness within the preceding three months prior, or those undergoing chronic treatments that could interfere with study objectives were excluded.

Participants meeting the inclusion criteria were selected after obtaining parental/legal guardian consent. They underwent interviews collecting epidemiological, clinical, and anthropometric data. Analytical variables were measured only in children classified as overweight (BMI between the 85th and 95th percentiles for age and sex) or obese (BMI > 95th percentile). Height was measured to the nearest millimeter using a portable stadiometer, while weight was recorded to the nearest 0.1 kg. Body mass index (BMI) was calculated using the formula BMI = weight in kilograms divided by height in meters squared, kg/m^2^.

### 2.4. Biochemical Data

Blood samples were collected from individuals meeting the criteria for overweight or obesity in the morning after a fasting period of more than 8 h, ensuring standardized conditions and minimizing potential circadian rhythm effects. All samples were processed and analyzed in the same laboratory to ensure consistency and minimize variability. Samples were centrifuged and stored at −80 °C until analysis. Serum concentrations of IL-6, TNF-α, adiponectin, and leptin were measured using sandwich enzyme-linked immunosorbent assay (ELISA) kits from R&D Systems, while Klotho levels were assessed with ELISA kits supplied by Abyntec Biopharma.

### 2.5. Statistical Analysis

Descriptive statistics were applied for variable analysis, with means and standard deviations (or ranges) reported for continuous variables and frequencies for categorical variables. The Shapiro–Wilk test was utilized to evaluate the normality of data distribution. Accordingly, parametric methods (Pearson’s rr) or non-parametric methods (Spearman’s ρρ) were applied as appropriate, based on the distribution properties of each variable (Supplementary Materials, Table S1). We also analyzed the correlations between various biomarkers (IL-6, TNF-α, leptin, adiponectin, leptin/adiponectin ratio, and Klotho) and BMI. Statistical significance was set at *p*-value < 0.05. Analyses were performed using GraphPad Prism software (v. 10.0; GraphPad, San Diego, CA, USA).

## 3. Results

A total of 340 patients were included in this study, of whom 276 (81.2%) met the criteria for normal weight and 64 (18.8%) for overweight or obesity (obese group). Within the latter group, the prevalence of overweight was 11.4% (39 patients) and obesity was 7.3% (25 patients).

The mean age (± standard deviation) was 11.80 ± 1.14 years in the non-obese group and 11.67 ± 1.08 years in the obese group, with no significant difference observed between the two groups (*p* = 0.40). Among the obese group, a significant difference was observed in sex distribution, with a higher proportion of males in the obese group (59.4%) than in the non-obese group (45.7%, *p* = 0.048). The percentage of participants born in Spain was similar between groups (96.9% in the obese group vs. 96.7% in the non-obese group, *p* = 0.95). Additionally, 34.4% of obese participants lived in urban areas with populations exceeding 10,000 inhabitants, compared to 31.5% in the non-obese group (*p* = 0.66).

Anthropometric characteristics at birth were also analyzed. The mean birth weight was significantly higher in the obese group (3.40 ± 0.44 g) compared to the non-obese group (3.18 ± 0.52 g, *p* = 0.003). However, no significant difference was observed in birth length, with a mean of 51.87 ± 15.97 cm in the obese group and 49.30 ± 2.48 cm in the non-obese group (*p* = 0.24).

Anthropometric and physiological characteristics were also analyzed. The mean current weight did not differ significantly between groups, at 45.06 ± 13.56 g in the obese group and 43.37 ± 10.64 g in the non-obese group (*p* = 0.35). However, current height was significantly greater in the obese group (153.12 ± 7.88 cm) compared to the non-obese group (148.94 ± 16.35 cm, *p* = 0.04). BMI was significantly higher in the obese group (26.17 ± 2.26 kg/m^2^) compared to the non-obese group (18.91 ± 2.36 kg/m^2^, *p* < 0.0001). Abdominal circumference was markedly higher in the obese group (87.05 ± 9.26 cm) compared to the non-obese group (68.60 ± 8.33 cm, *p* < 0.0001). While systolic blood pressure showed a trend toward higher values in the obese group (101.77 ± 23.88 mmHg) compared to the non-obese group (97.16 ± 15.88 mmHg), the difference was not statistically significant (*p* = 0.06). Similarly, diastolic blood pressure was slightly higher in the obese group (58.04 ± 15.31 mmHg) than in the non-obese group (55.46 ± 11.79 mmHg), but this difference did not reach statistical significance (*p* = 0.14).

Of the 64 pubertal patients meeting the criteria for overweight or obesity, the opportunity to proceed to Phase 2 of the study was offered. A total of 39 patients (61%) agreed to continue. Serum sample from one child was excluded from biochemical and biomarkers analysis due to poor quality, compromising data reliability. Biochemical parameters, including glucose, total cholesterol, triglycerides, HDL-cholesterol, aspartate aminotransferase, and alanine aminotransferase (ALT), were within normal ranges. A sex-based comparison revealed significantly higher ALT levels in men (20.6 ± 5.6 UI/L vs. 16.6 ± 5.3 UI/L; *p* = 0.03).

Additionally, four patients had insufficient serum volume for biomarker analysis.

**Figure 1:**
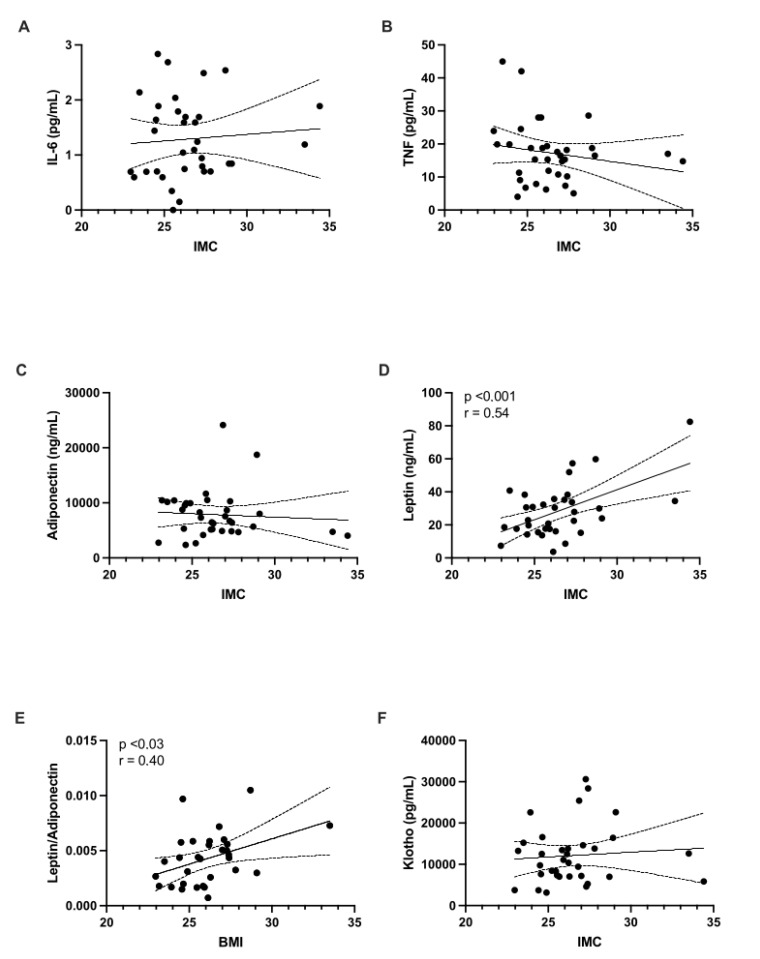
The relationship between body mass index (BMI) and the levels of the following biomarkers was analyzed across the entire study population: (**A**) IL-6; (**B**) TNF-α; (**C**) adiponectin; (**D**) leptin; (**E**) leptin/adiponectin ratio; and (**F**) Klotho.

**Figure 2:**
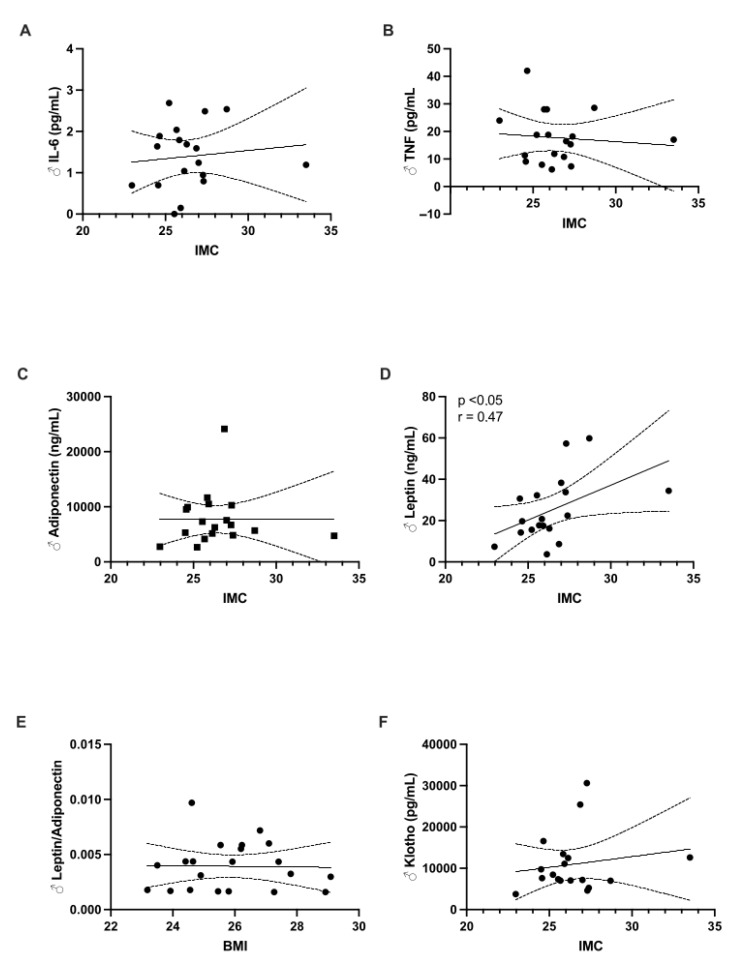
The relationship between body mass index (BMI) and the levels of the following biomarkers was analyzed exclusively in male children: (**A**) IL-6; (**B**) TNF-α; (**C**) adiponectin; (**D**) leptin; (**E**) leptin/adiponectin ratio; and (**F**) Klotho.

**Figure 3:**
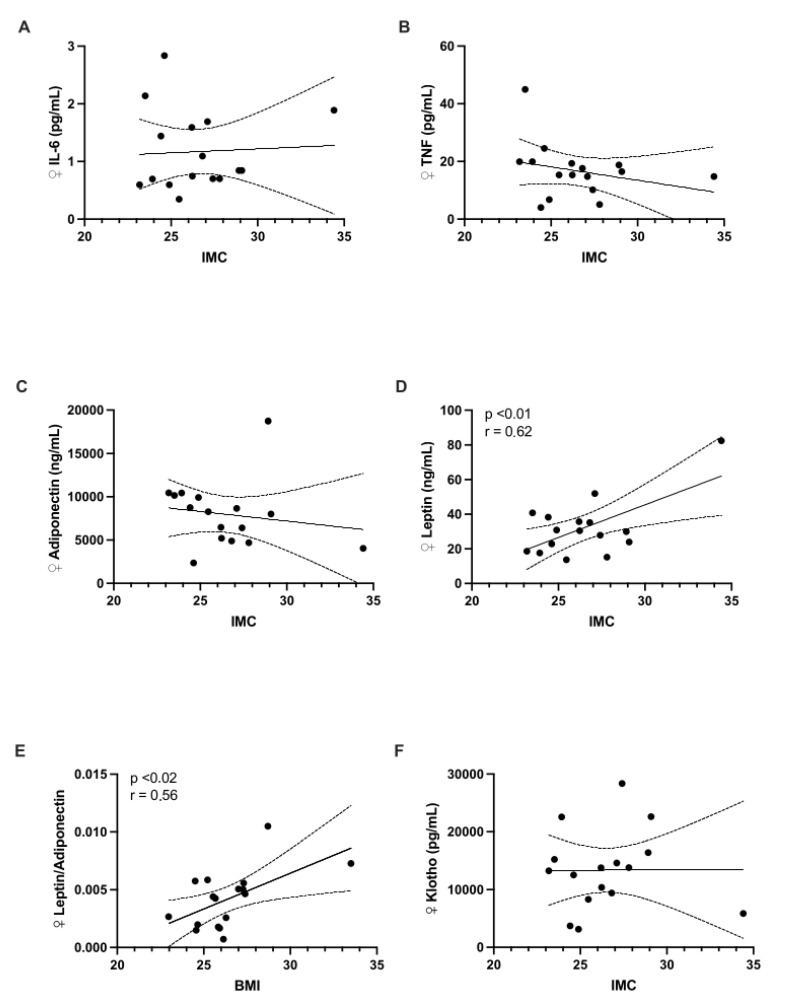
The relationship between body mass index (BMI) and the levels of the following biomarkers was analyzed exclusively in female children: (**A**) IL-6; (**B**) TNF-α; (**C**) adiponectin; (**D**) leptin; (**E**) leptin/adiponectin ratio; and (**F**) Klotho.

When comparing the different biomarkers by sex, no differences were observed (Table 1). After analyzing all samples (Figure 1), a significant positive correlation was observed between BMI and leptin levels (r = 0.54, *p* = 0.0008). A similar relationship was observed for the L/A ratio (r = 0.40, *p* = 0.025). When stratified by sex, significant correlations were found in females for leptin (r = 0.62, *p* = 0.009) and the L/A ratio (r = 0.56, *p* = 0.017) (Figure 2). In males, a significant correlation was observed only for leptin levels (r = 0.47, *p* = 0.044) (Figure 3).

## 4. Discussion

This study examined circulating levels of IL-6, TNF-α, adiponectin, leptin, the L/A ratio, and Klotho in obese children, contributing valuable data to the predominantly adult-focused existing literature. Notably, our findings highlight sex-specific associations between BMI and adipokine profiles.

Adiponectin and leptin are essential adipokines involved in metabolic regulation and have emerged as critical biomarkers for metabolic pathologies [31,32]. Leptin, in particular, is implicated in the upregulation of inflammatory cytokines such as IL-6 and TNF-α, contributing to the low-grade inflammation characteristics of obesity. This chronic inflammation is a key factor in the pathophysiology of metabolic disorders and cardiovascular diseases [18]. Several studies highlight the relationship between leptin and BMI and inflammation. For instance, Vales-Villamarin et al. [33] reported a significant positive correlation between BMI and leptin levels, as well as between BMI and hs-CRP concentrations in prepubertal children aged 6–8 years. However, after adjusting for leptin levels, the BMI–hs-CRP correlation was no longer significant in this age group. Conversely, in adolescents aged 12–17 years, BMI remained significantly correlated with both leptin and hs-CRP levels, even after leptin adjustment. These findings suggest that leptin mediates the BMI–hs-CRP association in prepubertal children, suggesting its role in early low-grade inflammation. In adolescents, however, hs-CRP levels may be influenced by factors independent of leptin. Notably, girls tend to have higher leptin levels than boys, likely due to hormonal differences and body composition. Our study confirmed a significant positive correlation between BMI and leptin levels in both male and female children. However, no significant differences in adiponectin levels were observed, either overall or stratified by sex. This finding contrasts with previous studies that have consistently demonstrated an inverse relationship between BMI and adiponectin levels in both adults and children [34,35]. Elevated adiponectin levels enhance insulin sensitivity by promoting glucose uptake in peripheral tissues and reducing glycogenolysis and hepatic gluconeogenesis [36]. A Danish study in healthy schoolchildren (8–17 years) found that girls showed significantly higher values of leptin and adiponectin levels than boys [31]. Additionally, girls showed a lower L/A ratio, which is a sensitive biomarker for early metabolic dysregulation [22,32]. In another Danish study involving children aged 6–18 years with normal weight or overweight/obesity, those with overweight/obesity exhibited had higher leptin, lower adiponectin, and elevated L/A ratios compared to their normal-weight peers [32]. Within this group, girls and boys in the highest L/A ratio quartile had a significantly greater likelihood of insulin resistance [32]. Regardless of obesity degree, leptin, adiponectin, and the L/A ratio were strongly associated with insulin resistance and cardiometabolic risks, with the L/A ratio demonstrating the strongest associations [32]. In a cohort of Italian prepubertal children and adolescents (aged 5–13 years), leptin and the L/A ratio increased significantly with BMI in both sexes, while adiponectin was significantly lower in overweight and obese girls but remained unchanged in boys [37]. Leptin independently predicted HOMA-IR in girls but not boys after adjusting for age, BM, lipids, and inflammatory mediators [37]. In our study, the lack of significant differences in certain biomarkers, such as adiponectin, in the overall analysis or when stratified by sex, may be explained by a combination of biological, population, and methodological factors [31,37].

IL-6 and TNF-α have been implicated in the increased risk of developing type 2 diabetes mellitus and atherosclerosis and exhibit an inverse relationship with adiponectin levels [38]. A prior study in school-aged children reported elevated IL-6 levels in individuals with higher BMIs (*p* < 0.05) [39]. However, we did not observe this association in our study, suggesting that the relationship between BMI and these pro-inflammatory cytokines may be influenced by other confounding factors or population-specific characteristics.

The role of Klotho in pediatric obesity remains insufficiently understood. A cross-sectional study analyzing Klotho serum levels in hospitalized children and adolescents aged 6–17 years reported significantly higher Klotho concentrations in obese children compared to their overweight and normal-weight peers, as well as in insulin-resistant compared to insulin-sensitive ones [28]. The hospitalized status of the participants represents a notable difference from our study, which focused on a non-hospitalized cohort. In another study investigating plasma Klotho levels in 11 obese patients, 12 individuals with restrictive anorexia nervosa (r-AN), and 11 controls, Klotho levels were significantly lower in both the obesity and r-AN groups compared to controls, with levels increasing markedly following BMI recovery in r-AN patients [40]. These findings suggest that Klotho may serve as a marker of normal nutritional status. Consistently, our study also did not observe significant differences in Klotho levels, further supporting its potential role as a nutritional biomarker rather than one directly associated with BMI or adiposity. In a longitudinal cohort study of school-age children (mean age 8.5 ± 1.8 years at baseline) followed over 4 years [29], negative associations between Klotho and BMI, waist circumference, body fat, visceral fat, HOMA-IR, and C-reactive protein were observed in girls but not boys, particularly among those with weight gain. In girls with weight gain, higher baseline Klotho levels were associated with reduced follow-up BMI, waist circumference, and visceral fat, suggesting a potential protective role of Klotho against visceral fat accumulation and metabolic risk. The absence of differences observed in our study may be attributed to its cross-sectional design and the older age of the participants, emphasizing the need for further research to clarify the role of Klotho in different pediatric populations and study settings.

This study has several limitations. First, only a subset of biomarkers was analyzed, selected for their relevance to the study population, to address gaps in the existing literature. Second, the small sample size and the absence of a control group, excluded for ethical reasons, may limit the generalizability of the findings. Conducting analyses on healthy children was considered unethical, as the procedures posed unnecessary risks without direct health benefits. Ethical guidelines emphasize minimizing harm and ensuring that research directly benefits participants, especially in vulnerable populations such as children [41]. Focusing exclusively on children with obesity provides data more relevant to understanding the condition and developing targeted, effective interventions. Third, this study was conducted in a single autonomous community, representing a localized sample, which may restrict the applicability of the results to broader populations. Fourth, the cross-sectional design of this study precludes the establishment of causal relationships. A final limitation of this study is that it exclusively analyzes a Spanish population, which may restrict the extrapolation of the findings to other populations with different genetic, environmental, or cultural characteristics [42]. Despite these limitations, this study underscores the importance of biomarkers such as leptin and adiponectin in understanding the pathophysiology of childhood obesity. These biomarkers not only elucidate underlying mechanisms but also hold promise as tools for public health interventions, including identifying at-risk populations, monitoring intervention outcomes, and tailoring strategies to individual needs. Future research should focus on integrating these biomarkers into public health frameworks to enhance the effectiveness of obesity prevention and management programs.

In summary, this study, conducted in children aged 10–14 years, found that increased serum leptin concentrations and L/A ratios correlate with higher BMI, but only in females. No significant associations were observed between BMI and adiponectin, IL-6, TNF-α, or Klotho levels. These findings underscore the complexity of the relationship between obesity and metabolic markers in children and highlight the need for further longitudinal research to elucidate these associations and their potential clinical implications.

## Figures and Tables

**Table 1:** Summary of biomarker levels: minimum, 25th percentile, and 75th percentile.

	IL-6 (pg/mL)	TNF (pg/mL)	Leptin (pg/mL)	Adiponectin (ng/mL)	L/A Ratio	Klotho (pg/mL)
All the samples						
25% Percentile	0.70	10.33	16.85	4844	0.0019	7032
Median	1.14	15.90	23.92	6594	0.0043	10.731
75% Percentile	1.81	19.20	34.81	9834	0.0057	14.758
Male Only (*n* = 18)						
25% Percentile	0.77	10.33	15.22	4803	0.0017	7010
Median	1.41	16.76	20.24	6488	0.0043	8034
75% Percentile	1.92	24.97	33.95	10,030	0.0056	12,828
Female Only (*n* = 16)						
25% Percentile	0.69	11.33	15.22	4977	0.0020	8583
Median	0.84	15.90	20.24	8146	0.0041	13,524
75% Percentile	1.60	19.77	33.95	10,100	0.0059	16,122

## Data Availability

The materials described in this manuscript will be made available upon reasonable request.

## Supplementary Materials

The following supporting information can be downloaded at: https://www.mdpi.com/article/10.3390/children12010089/s1, Table S1: Details of the Biomarkers assay methods.
