# Supplementary material for: Cross-Sectional Analysis of IL-6, TNF-α, Adiponectin, Leptin, and Klotho Serum Levels in Relation to BMI Among Overweight and Obese Children Aged 10–14 in La Rioja, Spain
Source: Children (Basel). 2025 Jan 14;12(1):89. doi: 10.3390/children12010089 (PMC11763806; doi:10.3390/children12010089)
Supplement: Supplementary file 1 [file children-12-00089-s001.zip › children-3397990-supplementary.pdf]

Table S1. Details of the Biomarkers assay methods.

Biomarker	Procedure	Minimum Detectable Dose (MDD)	Commercial
IL-6	Quantikine® ELISA	0.368 pg/ml	R&D Systems
TNF-α	Quantikine® ELISA	4 pg/ml	R&D Systems
Leptin	Quantikine® ELISA	7,8 pg/ml	R&D Systems
Adiponectin	Quantikine® ELISA	0,195 ng/ml	R&D Systems
Klotho	High sensitive ELISA	6,4 pg/ml	Abyntek

Note: IL-6 = interleukin 6; TNF = Tumor necrosis factor; L/A = leptin/adiponectin ratio.
